# *GmCLC1* Confers Enhanced Salt Tolerance through Regulating Chloride Accumulation in Soybean

**DOI:** 10.3389/fpls.2016.01082

**Published:** 2016-07-25

**Authors:** Peipei Wei, Longchao Wang, Ailin Liu, Bingjun Yu, Hon-Ming Lam

**Affiliations:** ^1^College of Life Sciences, Nanjing Agricultural UniversityNanjing, China; ^2^Center for Soybean Research of the Partner State Key Laboratory of Agrobiotechnology and School of Life Sciences, The Chinese University of Hong KongHong Kong, China

**Keywords:** chloride transporter, Cl^-^ toxicity, *GmCLC1*, salt stress, soybean root transformation, transgenic *Arabidopsis*

## Abstract

The family of chloride channel proteins that mediate Cl^-^ transportation play vital roles in plant nutrient supply, cellular action potential and turgor pressure adjustment, stomatal movement, hormone signal recognition and transduction, Cl^-^ homeostasis, and abiotic and biotic stress tolerance. The anionic toxicity, mainly caused by chloride ions (Cl^-^), on plants under salt stress remains poorly understood. In this work, we investigated the function of soybean Cl^-^/H^+^ antiporter GmCLC1 under salt stress in transgenic *Arabidopsis thaliana*, soybean, and yeast. We found that *GmCLC1* enhanced salt tolerance in transgenic *A. thaliana* by reducing the Cl^-^ accumulation in shoots and hence released the negative impact of salt stress on plant growth. Overexpression of *GmCLC1* in the hairy roots of soybean sequestered more Cl^-^ in their roots and transferred less Cl^-^ to their shoots, leading to lower relative electrolyte leakage values in the roots and leaves. When either the soybean *GmCLC1* or the yeast chloride transporter gene, *GEF1*, was transformed into the yeast *gef1* mutant, and then treated with different chloride salts (MnCl_2_, KCl, NaCl), enhanced survival rate was observed. The result indicates that *GmCLC1* and *GEF1* exerted similar effects on alleviating the stress of diverse chloride salts on the yeast *gef1* mutant. Together, this work suggests a protective function of GmCLC1 under Cl^-^ stress.

## Introduction

Ionic stress, osmotic stress, nutritional imbalance, and oxidative damage are the main causes for salt injury of plants, among which ionic stress is the primary factor (Xiao et al., 2013; Adem et al., 2014). Sodium chloride is the most common salt in environment. In salinized conditions, salt changed the water potential around the root first causing the osmotic stress, then inducing ionic stress by the accumulation of Na^+^ and Cl^-^ (Deinlein et al., 2014; Gupta and Huang, 2014; Nguyen et al., 2015; Nie et al., 2015). Plants counter these stresses through strategies such as extracellular exclusion of excess ions across the plasma membrane or intracellular vacuolar compartmentalization to reduce the effective Na^+^ and Cl^-^ levels inside the cell, especially in the aerial parts (Zhang et al., 2011; Hasegawa, 2013). In general, crops such as cotton, rice, and barley are more sensitive to Na^+^ than Cl^-^ (Qu et al., 2009). So far, the researches on plant physiological and molecular mechanisms of salt tolerance have mostly focused on cation (Na^+^) poisoning and adaptations, such as the NSCCs (non-selective cation channels) and HKTs (High-affinity K^+^ Transporter), both controlling Na^+^ import into the cell, together with Na^+^/H^+^ antiporters, such as AtSOS1 (*Arabidopsis* Salt Overly Sensitive 1) located in the plasma membrane for excluding Na^+^ from cells, and AtNHX1 (*Arabidopsis* vacuolar Na^+^/H^+^ antiporter) located in the tonoplast for compartmentalizing Na^+^ mainly in the vacuoles (Adams and Shin, 2014; Deinlein et al., 2014; Nguyen et al., 2015). However, salt injuries to plants resulting from anions (mainly Cl^-^) have long been under-investigated.

Cl^-^ is the main form of anions in plant cells besides nitrate (${\mathrm{NO}}_{3}^{–}$). As one of the essential micronutrient elements for higher plant growth and development, Cl^-^ is involved in photosynthesis, stomatal movement, cellular osmotic pressure maintenance, charge balance, and disease resistance (Jossier et al., 2010; Tealle and Tyerman, 2010; Guo et al., 2014). Excessive amount of Cl^-^ lead to various adverse effects, such as negatively impacting the absorption of macronutrient elements (nitrogen [N], phosphorus [P], and potassium [K]), leaf water potential, causing stomatal closure and the accumulation of reactive oxygen species (ROS) in chloroplasts, which severely affected crop quality and yield (Yu and Liu, 2004; Nguyen et al., 2015). Some studies reported that certain crops or woody species such as tobacco, tomato, barley, grapevine, citrus, *Glycine max, Lotus*, and poplar were more sensitive to Cl^-^ than to Na^+^ under salt stress (Moya et al., 2003; Luo et al., 2005; Chen and Yu, 2007; Brumós et al., 2010; Tregeagle et al., 2010). The genetic differences in the control of Cl^-^ transport from roots to shoots or the ability to maintain a low shoot Cl^-^ level is the key determinant of Cl^-^/salt tolerance (Zhang et al., 2011; Henderson et al., 2014). The anion channels or transporters, widely distributed in all types of organelle membranes (such as plasma membrane, tonoplast, endoplasmic reticulum, mitochondria, and chloroplasts, etc.), predominantly mediate Cl^-^ and ${\mathrm{NO}}_{3}^{–}$ flux across the membrane, playing a vital function in plant nutrition absorption and transportation, adjustment of cellular action potential and turgor pressure, stomatal movement, hormone signal recognition and transduction, Cl^-^ homeostasis under abiotic (salt, heavy metal, low temperature, etc.) or biotic stress conditions (Jossier et al., 2010; Barbier-Brygo et al., 2011; Wei et al., 2013; Guo et al., 2014; Nguyen et al., 2015). Among them, the *CLCs* (Chloride channel proteins) family mainly mediate Cl^-^ transport and have attracted the most research interests (Zifarelli and Pusch, 2010). Currently, plant *CLCs* have been identified in *Arabidopsis*, tobacco, rice, potato, corn, spinach, citrus, salt cress, soybean, and maize (Wei et al., 2013; Zhou et al., 2013; Li et al., 2014; Wang et al., 2015). In response to Cl^-^ toxicity under salt stress, the NaCl-treated plants utilize the anion transporters such as CLCs to adjust and reduce Cl^-^ accumulation in the cell cytoplasm. In soybean, Li et al. (2006) reported that the protein encoded by the salt- and polyethylene glycol-inducible *GmCLC1* gene (GenBank accession: AY972079, or Phytozome database: *Glyma05g14760*) localizes on tonoplast and can transport and sequester Cl^-^ into the vacuoles of plant cells. Wong et al. (2013) further found that the transmembrane Cl^-^ transfer activity of the GmCLC1 protein depends on the cytoplasmic pH value, suggesting that it is most likely a kind of Cl^-^/H^+^ antiporter that participates in the maintenance of intracellular Cl^-^ homeostasis and regulates Cl^-^/salt tolerance.

In the current study, we further investigated the functions of how *GmCLC1* regulates Cl^-^ transportation in salt-stressed plants and yeast. We found that overexpressing *GmCLC1* in *Arabidopsis thaliana*, soybean hairy roots and composite plants, as well as yeast mutant could enhance salt tolerance by regulation Cl^-^ homeostasis.

## Materials and Methods

### Plant Materials, Bacteria and Yeast Strains, and Plasmids

Plant seeds including wild-type (WT) *A. thaliana* (Columbia ecotype glabrous1), *G. max* (L.) Merr. cultivars Jackson (salt-sensitive), Lee68 (salt-tolerant) and N23674 (salt-tolerant), *Escherichia coli* DH5α, *Agrobacterium tumefaciens* strain GV3101, *Agrobacterium rhizogenes* strain K599, binary vector for plant transformation pCAMBIA1300, the yeast *Δgef1* mutant (derived from *Saccharomyces cerevisiae* BY4741) and yeast expression plasmid pYES2 were used in this study.

### *GmCLC1* Gene Cloning and Vectors Construction

The seeds of *G. max* N23674 cultivar were surface-sterilized with 1 g dm^-3^ HgCl_2_ for 5 min, then fully rinsed in distilled water, soaked in distilled water for 6 h, and finally germinated at 25°C in the dark. The germinated seeds were grown on vermiculite irrigated with 1/2 Hoagland solution in a greenhouse with temperature at 25 ± 2°C and humidity ranging from 60 to 70%. Total RNA was extracted from 10-days-old seedlings using the Trizol reagent (Invitrogen, USA). First-strand cDNAs were synthesized with 2 μg total RNAs using a PrimeScript^TM^ II 1st Strand cDNA Synthesis Kit (TaKaRa, Dalian) according to the manufacturer’s protocol. The full-length coding region of *GmCLC1* was amplified from the cDNA using the following PCR protocol: 94°C 3 min; 30 cycles of 94°C 30 s, 55°C 30 s, and 72°C 2 min 30 s; and 72°C 5 min in a 25 μL reaction mixture [2 μL of first strand cDNA, 0.15 mM MgCl_2_, 0.2 mM dNTPs, 0.4 μM of each primer, 0.25 U Taq DNA polymerase (TaKaRa, Dalian) and 10× PCR buffer]. Primers used: 5′-ATGGGTGAGGAATCCAGTTT-3′ and 5′-CTTCCTCTTTGATTTTGCCAG-3′. The PCR products were then cloned into pMD19-T vector (TaKaRa, Dalian) for sequencing.

Subsequently, the open reading frame of *GmCLC1* was amplified from cDNA by PCR (95°C 3 min, 28 cycles of 95°C for 30 s, 55°C for 30 s, and 68°C for 2.5 min) with KOD-Plus (TOYOBO, Japan), and ligated into the plasmid pCAMBIA1300 or pYES2 to obtain the recombinant plasmid pCAMBIA1300-*GmCLC1* (primers used: 5′-GGGGTACCATGGGTGAGGAATCCAGTTT-3′ and 5′-GGACTAGTCTTCCTCTTTGATTTTGCCAG-3′) or pYES2-*GmCLC1* (primers used: 5′-CCGGAATTCATGGGTGAGGAATCCAG-3′ and 5′-CCGCTCGAGTCACTTCCTCTTTGATTTTG-3′). After sequence verification, the recombinant plasmid pCAMBIA1300-*GmCLC1* was transformed into *A. tumefaciens* GV3101 or *A. rhizogenes* K599, respectively.

### Seed Germination and Root Elongation Experiments with WT and *GmCLC1*-Transgenic *A. thaliana*

The binary vector pCambia1300-*GmCLC1* was transformed into WT *Arabidopsis* plants using the floral dip method mediated by *A. tumefaciens* (Clough and Bent, 1998). Transformants were selected by germination on Murashige and Skoog (MS) agar medium supplemented with 40 mg L^-1^ hygromycin B. Homozygous lines (L_1_–L_5_) of T_2_ plants were identified by PCR. The seeds of *A. thaliana* WT and a homozygous *GmCLC1*-transgenic line (L_1_) were sterilized and sown evenly on MS agar medium (pH = 5.8; Nie et al., 2015) containing 0, 150, and 200 mM NaCl, respectively. The agar plates were put in an illuminated growth chamber under a 14 h light/10 h dark cycle at 20 ± 2°C, with 60–70% relative humidity. The seed germination percentage (%) was recorded after 7 days. For measuring the root elongation of *A. thaliana* under salt stress, seeds of WT and *GmCLC1*-transgenic plants were cultured on MS agar medium (pH = 5.8) without NaCl for 5 days. Then seedlings with similar root lengths were selected and transferred onto MS agar medium (pH = 5.8) containing 0, 150, and 200 mM NaCl. After placing the agar plates vertically for 5 days, the seedlings of WT and *GmCLC1*-transgenics were photographed and the root lengths were measured.

### Determination of Leaf Relative Water Content (RWC) and Chlorophyll Fluorescence (Fv/Fm), Root and Leaf Relative Electrolyte Leakage (REL) of WT and *GmCLC1*-Transgenic *A. thaliana*

Seeds of *A. thaliana* WT and *GmCLC1*-transgenic line (L_1_) were sterilized and sown on MS agar medium (pH = 5.8), and then placed in a growth chamber under a 14 h light/10 h dark cycle at 20 ± 2°C, with 60–70% relative humidity. After 8 days, the seedlings were then transferred to pots containing a sterilized peat moss and vermiculite mixture and grown for 5 days. Then the seedlings were treated with increasing concentrations of NaCl (50 mM NaCl for 2 days, 100 mM NaCl for the next 2 days, 150 mM NaCl solution for another 7 days). Finally, leaf relative water content (RWC) was measured according to the method described by Hu et al. (2016). Leaf chlorophyll fluorescence (Fv/Fm) was measured at room temperature with a plant efficiency analyzer (Handy PEA Fluorometer, Hansatech Instruments, UK; (Tian et al., 2014). Root or leaf relative electrolyte leakage (REL) was assayed using the method described by Hu et al. (2016) with a digital conductivity meter (DDS-307, Shanghai, China).

### Analyses of Cl^-^ Contents in Roots and Shoots of WT and *GmCLC1*-Transgenic *A. thaliana* Seedlings

Seeds of WT and *GmCLC1*-transgenic *A. thaliana* were sterilized and sown in pots containing a sterilized peat moss and vermiculite mixture and grown in a growth chamber under a 14 h light/10 h dark cycle at 20 ± 2°C, with 60–70% relative humidity. After 30 days, plants were treated with 1/2 X Hoagland solution containing 150 mM NaCl for 0, 4, 12, 24 h, 2, 4, and 6 days, respectively. Then the roots and shoots of *Arabidopsis* seedlings were sampled and Cl^-^ contents were measured with the method described (Zhou and Yu, 2009).

### Assays of Maximum Lengths and Fresh Weights of the Hairy Roots of Transformed Soybean Cotyledons, and REL and Cl^-^ Contents of Soybean Hairy Roots Composite Plants

Hairy root transformation was performed according to Ali et al. (2012). *A. rhizogenes* strain K599 containing the recombinant binary vector pCAMBIA1300-*GmCLC1* was grown in yeast extract peptone (YEP) medium containing 50 mg/L ampicillin (Amp) and 200 μM acetosyringone at 28°C for 16 h. Then the bacterial culture was centrifuged, and the pellet was resuspended gently in 10 mM MgSO_4_ solution followed by two washings, and adjusted to OD_600_ ≈ 0.5.

The cotyledons of soybean (Jackson and Lee68 cultivars) were scored with a scalpel and the wounds were infected with the pCAMBIA-containing *A. rhizogenes* infection solution for 1 h in dark at room temperature, and then transferred to moist filter paper and incubated in the dark for 5 days (at 25 ± 2°C). After that, the infected cotyledons were transferred to a growth chamber under a 12 h light/12 h dark cycle at 25 ± 2°C. New hair roots that sprout from the infected cotyledons that were free of *Agrobacterium* as screened by PCR and at similar lengths were selected and transferred into 1/2 X Hoagland solution (pH = 6.5) containing 0, 100, and 150 mM NaCl, respectively Hairy roots infected with *A. rhizogenes* strain K599 containing the empty vector pCAMBIA1300 served as the control. After 5 days treatment, hairy root growth (maximum root length and root fresh weight) was photographed and measured (Qi et al., 2014).

For whole-plant transformation, surface-sterilized soybean seeds (Jackson and Lee68 cultivars) were sown in pots containing a sterilized peat moss and vermiculite mixture. When the first pair of true leaves had fully expanded, the cotyledon nodes of the soybean seedlings were infected with the *A. rhizogenes* strain K599 infection solution for 1 h at room temperature by injection. Then the wounds were covered with moist vermiculite and incubated for 5 days at 28°C under a 12 h light/12 h dark cycle. After 7 days, hairy root lines that were free of *Agrobacterium* as screened by PCR were selected and the original roots removed from the seedlings. These seedlings were then cultured in 1/2 X Hoagland solution. Seedlings infected with *A. rhizogenes* strain K599 containing the empty vector pCAMBIA1300 served as the negative control. After 10 days, Jackson and Lee68 seedlings with similar-length hairy roots were selected and treated with 1/2 X Hoagland solution containing 120 mM NaCl for 3 and 5 days, respectively, and seedlings grown in Hoagland solution without NaCl served as the untreated control. All the solutions listed above were replaced every 3 days throughout the experiments. REL and Cl^-^ contents in roots, stems, first true leaves, and the first and second trifoliates were measured accordingly.

### Tolerance Tests Using the *Δgef1* Yeast Mutant

The yeast expression vector pYES2-*GmCLC1* was transformed into *Δgef1* mutant yeast (*S. cerevisiae*) using the PEG/LiAc procedure and transformants were screened by PCR (Gietz, 2014). Ten-fold serial dilutions (starting at OD_550_ ≈ 0.5) of each yeast culture were plated on agar supplemented with YPD medium (1% yeast extract/2% peptone/2% dextrose), YPG medium (1% yeast extract/2% peptone/2% galactose), or YPG medium supplemented with 1 M NaCl, 1 M KCl, or 3 mM MnCl_2_. Plates were then incubated at 30°C and photographs were taken after 60 h. For Cl^-^ content measurements, the above-mentioned yeast cells were grown in the liquid YPG medium plus 1.0 M NaCl, and collected during the exponential growth phase (OD_550_ ≈ 0.2), and their Cl^-^ contents were assayed by AgNO_3_ titration as described by Zhou and Yu (2009).

### Statistical Analyses

Data were expressed as mean ± SD of three independent experiments and were analyzed using one-way analysis of variance by SPSS 19.0, and pairwise comparisons were performed using Duncan’s test.

## Results

### *GmCLC1* Alleviates NaCl Stress on Transgenic *A. thaliana* by Reducing Cl^-^ Accumulation in Leaves

**FIGURE 1:**
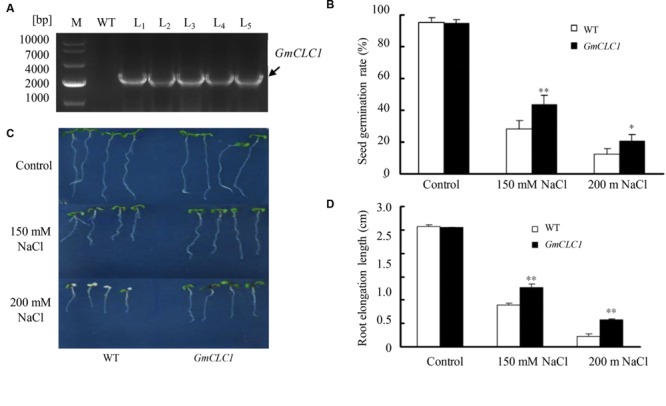
**Growth characteristics of *GmCLC1*-transgenic *Arabidopsis thaliana*. (A)** Genotyping by PCR of *GmCLC1*-transgenic *A. thaliana* lines (L_1_–L_5_) to confirm success in transformation. All five lines contain the transgene. Comparison in **(B)** seed germination rate, **(C)** seedlings growth, and **(D)** root lengths between *A. thaliana* wild-type (WT) and transgenic plants (*GmCLC1*; using L_1_ as the representative) sown on MS agar medium containing different concentrations of NaCl. **(B)** Seeds of WT and *GmCLC1* (30 seeds per plate each with three replicates) were surface-sterilized and stratified at 4°C for 2–4 days and then sown on MS medium supplemented with 150 or 200 mM NaCl. After 7 days of incubation, the seed germination rate (%) was measured. **(C,D)** 5-days-old WT and *GmCLC1* with nearly equal root lengths were transferred to MS agar medium supplemented with 0, 150, or 200 mM NaCl. After 5 days of vertical incubation, the seedlings’ growth phenotypes were photographed and the root lengths measured. M: DNA markers. ^∗^/^∗∗^: The difference between WT and *GmCLC1* was significant at *p* < 0.05 and *p* < 0.01 respectively, using Duncan’s test. Each bar represents the mean and SD of for **(C)** 30 seeds per plate each with three replicates; for **(D)** more than 10 plants for each treatment.

The cDNA of *GmCLC1* was cloned from *G. max* cultivar N23674. The encoded protein product has the same sequence as the annotated product of *Glyma05g14760*. We have constructed five independent transgenic lines expressing *GmCLC1*. All transgenic lines exhibited NaCl tolerance (**Supplementary Figure S1**) and hence we selected one typical line for detailed analysis. The *A. thaliana* plant was successfully transformed with *GmCLC1* as shown with genotyping by PCR (**Figure 1A**). When grown on plate containing 150 or 200 mM NaCl, the seed germination rates of both WT and *GmCLC1*-transgenic line (L_1_) declined as the NaCl concentration increased. However, under 150 or 200 mM NaCl treatments, the transgenic line exhibited a higher germination rate than WT (**Figure 1B**). Also, under 200 mM NaCl treatment, the seedling growth of WT and *GmCLC1*-transgenic line in both roots and shoot were inhibited compared to the untreated control (**Figure 1C**). However, the root elongation of the *GmCLC1*-transgenic line was significantly higher than those of WT under either 150 or 200 mM NaCl stress (*p* < 0.01; **Figure 1D**). This indicates that ectopic expression of *GmCLC1* in *A. thaliana* could enhance seed germination and seedling growth under salt stress.

**FIGURE 2:**
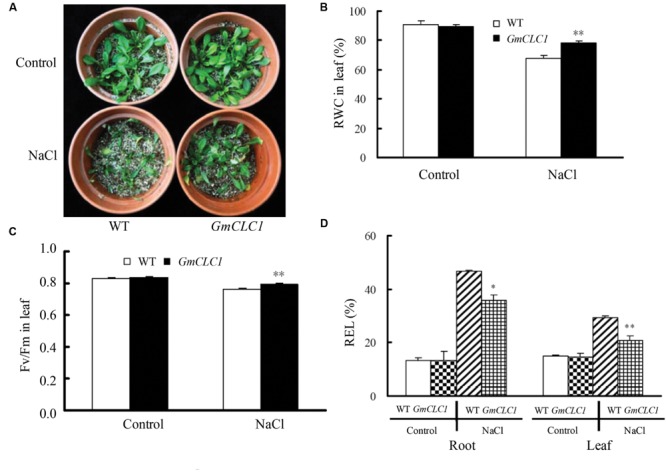
**Effects of NaCl treatment on **(A)** growth, **(B)** leaf relative water content (RWC), **(C)** chlorophyll fluorescence (Fv/Fm), and **(D)** relative electrolyte leakage (REL) in roots and leaves of *A. thaliana* WT and transgenic *GmCLC1* seedlings.** Seeds of *Arabidopsis* WT and transgenic *GmCLC1* plants were surface-sterilized and kept at 4°C for 2–4 days and then sown on MS medium. After 8 days of incubation, the seedlings were transferred into plastic pots filled with a sterilized peat moss and vermiculite mixture, and fertilized with ½-strength Hoagland nutrient solution for 5 days in the greenhouse. The nutrient solution was continuously replaced with half-strength Hoagland solution containing 50 mM NaCl for 2 days, followed by 100 mM NaCl for 2 days, and finally 150 mM NaCl for 7 days. No NaCl was added to the nutrient solution for the control treatment. ^∗^/^∗∗^: The difference between WT and *GmCLC1* was significant at *p* < 0.05/0.01, respectively, using Duncan’s test. Each bar represents the mean and SD of at least three replicates.

When WT and *GmCLC1*-transgenic seedlings were continuously exposed to NaCl stress with increasing concentrations (50 mM NaCl solution for 2 days, and 100 mM NaCl solution for next 2 days, followed by 150 mM NaCl solution for 7 days), the transgenic plants appeared to be healthier than WT (**Figure 2A**), and RWC and Fv/Fm were also significantly higher than those of WT (*p* < 0.01; **Figures 2B,C**), while the REL values were significantly lower than those of WT (*p* < 0.01 for root, *p* < 0.05 for leaf; **Figure 2D**). This further shows that the *GmCLC1*-transgenic *A. thaliana* plants suffered less leaf water loss, exhibited more stable photosynthetic capacity and had reduced salt injuries compared to untransformed WT when under salt stress.

**FIGURE 3:**
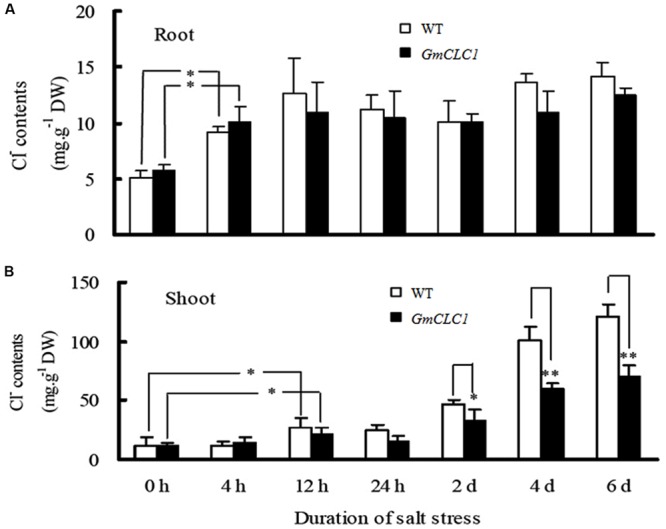
**Dynamic changes in the chloride (Cl^-^) contents in **(A)** roots and **(B)** shoots of *A. thaliana* WT and transgenic *GmCLC1* seedlings under 150 mM NaCl stress for 6 days.** Seeds of WT and *GmCLC1* transgenic *A. thaliana* were grown on pots containing the sterilized peat moss and vermiculite mixture and grown. After 30 days, plants were treated with 1/2 Hoagland solution containing 150 mM NaCl for 0, 4, 12, 24 h, 2, 4, and 6 days, respectively, then the roots **(A)** and shoot **(B)** of *Arabidopsis* seedlings were sampled and Cl^-^ contents were measured. ^∗^/^∗∗^: The difference between WT and the *GmCLC1* transgenic line was significant at *p* < 0.05/0.01, using Duncan’s test. Each bar represents the mean and SD of at least three replicates.

During the 6-days treatment with 150 mM NaCl, the Cl^-^ contents in the roots of both WT and *GmCLC1*-transgenic seedlings increased significantly within the first 4 h and then leveled off (**Figure 3A**). However, the Cl^-^ contents in the shoots of both WT and transgenic seedlings increased steadily throughout the duration of the experiment, with the increase in the shoots of WT being significantly higher than in the transgenic plants (*p* < 0.01; **Figure 3B**), indicating that *GmCLC1* reduces salt stress partly by reducing the Cl^-^ accumulation in shoots.

### *GmCLC1* Alleviates NaCl Stress on Transgenic Soybean Hairy Root Growth and Composite Plants

**FIGURE 4:**
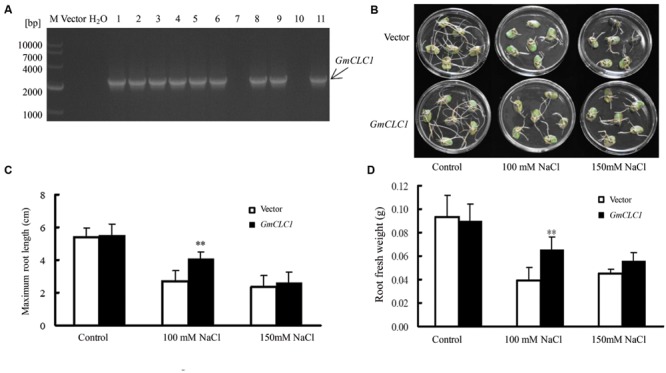
**Growth characteristics of transgenic soybean (hair root *GmCLC1*) under NaCl treatment. (A)** PCR verification of the presence of the transgene, *GmCLC1*, in the transgenic soybean hairy roots (*GmCLC1*) that had undergone *Agrobacterium rhizogenes*-mediated cotyledon transformation. Nine out of the 11 lines were successfully transformed. M: DNA marker; Vector: empty vector, as negative control; ddH_2_O: distilled deionized water, as negative control. Comparisons of **(B)** hairy root phenotype, **(C)** maximum root length, and **(D)** root fresh weight of empty vector-transformed control and *GmCLC1* grown in with 1/2 Hoagland solution containing 0, 100, and 150 mM NaCl for 5 days. Each bar represents the mean and SD of three replicates each with six soybean cotyledons. ^∗∗^: The difference between was significant at *p* < 0.01, using Duncan’s test.

The transformation of soybean cotyledon hairy roots with *GmCLC1* was shown to be successful through genotyping by PCR (**Figure 4A**). Under normal growing condition without additional NaCl, both empty vector- and *GmCLC1*-transformed hairy roots grew well, with no obvious difference (**Figure 4B**). However, under 100 mM NaCl stress, the growth of the hairy roots of both *GmCLC1*-transformed and empty vector-transformed lines were significantly inhibited, and the numbers of root branching were also reduced as compared to the untreated control. The maximum root lengths and fresh weights of empty vector-transformed hairy roots decreased more significantly than the *GmCLC1*-transformed ones (*p* < 0.01). At the NaCl concentration of 150 mM, the growth of the hairy roots of both *GmCLC1*- and empty vector-transformed cotyledons were inhibited, but there was no significant difference between the two (**Figures 4C,D**).

**FIGURE 5:**
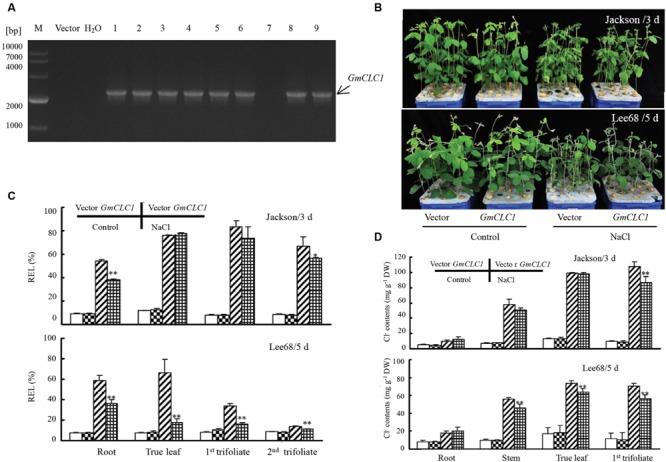
**Growth phenotype and physiological responses of soybean hairy root composite plants (*GmCLC1*) under NaCl treatment. (A)** PCR verification of the presence of the transgene, *GmCLC1*, in the soybean hairy root composite plants (lines 1–6 and 8–9). M: DNA markers; Vector: Empty vector-transformed plants (negative control); ddH_2_O: distilled deionized water (negative control). **(B)** Phenotypes of empty vector-transformed and *GmCLC1*-transgenic plants. Surface-sterilized soybean seeds were sown in container till the first pair of unifoliate leaves was fully expanded, the cotyledon node site of soybean seedlings were infected with the *A. rhizogenesin* K599 for 1 h at room temperature. The wound was then covered with moist vermiculite and co-cultivated with *A. rhizogenesin* K599 for 5 days at 25 ± 2°C under a 12/12 h light/dark cycle. After 7 days, hairy root lines were identified by PCR for free of bacterium. The original roots were removed and the seedlings with positive hairy roots were cultured in 1/2 Hoagland solution. After 10 days, seedlings were treated with 1/2 Hoagland solutions containing 120 mM NaCl for 3 or 5 days, respectively, and solutions without NaCl as the untreated control. **(C)** REL and **(D)** Cl^-^ content of *GmCLC1*-transgenic soybean plants versus empty vector-transformed controls (Vector) in different parts of the plant. Control: no NaCl was given to the growth medium; DW: dry weight; ^∗^/^∗∗^: The difference between was significant at *p* < 0.05/0.01, using Duncan’s test. Each bar represents the mean and SD of three replicates.

Building on the results we obtained using the *GmCLC1*-transgenic soybean cotyledon hairy root system, we further tested the response of whole *GmCLC1*-transgenic soybean hairy root composite plants to salt stress. First, the presence of the transgene in the soybean plants was confirmed by PCR (**Figure 5A**). Without any additional NaCl in the culture medium, there was no obvious phenotypic difference between the *GmCLC1*-transgenic soybean (including both Jackson and Lee68 cultivars) hairy root composite plants and the empty vector-transformed plants (**Figure 5B**). There was also no significant difference in the REL values of roots, first true leaves, the first and second trifoliate leaves among all the genotypes when grown in NaCl-free medium (**Figure 5C**).

When Jackson or Lee68 *GmCLC1*-transgenic soybean hairy root composite plants and the corresponding empty vector-transformed plants were exposed to 150 mM NaCl solution, the relatively salt-sensitive Jackson cultivar (both vector-only and *GmCLC1*) displayed obvious salt injury symptoms (with severely withered leaves) on the 3rd day, while the salt-tolerant Lee68 cultivar (both vector-only and *GmCLC1*) showed only mildly withered leaves up to the 5th day (**Figure 5B**). Furthermore, the hairy root composite plants of both cultivars that were transformed with *GmCLC1* showed better salt adaptation than their empty vector-transformed counterparts, especially for the more salt-tolerant Lee68 cultivar (**Figure 5B**). When examining the REL values in separate parts of the composite soybean plants, we found the REL values of roots, first true leaves, the first and second trifoliate leaves of *GmCLC1* were all significantly lower than their empty vector-transformed counterparts from the Lee68 cultivar (*p* < 0.01). The REL values of the second trifoliates of the *GmCLC1*-transgenic Lee68 cultivar, especially, were comparable to those of the water control. The REL values of the roots and second trifoliate leaves of *GmCLC1* of the Jackson cultivar were also significantly lower than their empty vector-transformed counterparts (*p* < 0.01 and 0.05, respectively; **Figure 5C**).

In addition, under salt stress, the Cl^-^ contents in roots, stems, first true leaves and the first trifoliate leaves of both Jackson and Lee68 hairy root composite plants (including both *GmCLC1* and vector-only) were dramatically increased compared to the water control. It is clear that the *GmCLC1*-transgenic plants of the Lee68 cultivar, when compared to their empty vector-transformed counterparts, had significantly lower Cl^-^ accumulation in their stems, first true leaves and the first trifoliate leaves (*p* < 0.01), and *GmCLC1*-transgenic plants of the Jackson cultivar also had significantly lower Cl^-^ accumulation in the first trifoliates (*p* < 0.01; **Figure 5D**). This indicates that the ectopic expression of *GmCLC1* in Jackson or Lee68 hairy root composite plants can reduce the Cl^-^ transportation and accumulation in the aerial parts of the plant, especially for the salt-tolerant Lee68 cultivar.

### *GmCLC1* Enhances Survival of the Chloride-Channel-Deficient Yeast Mutant *Δgef1* under MnCl_2_, NaCl, or KCl

**FIGURE 6:**
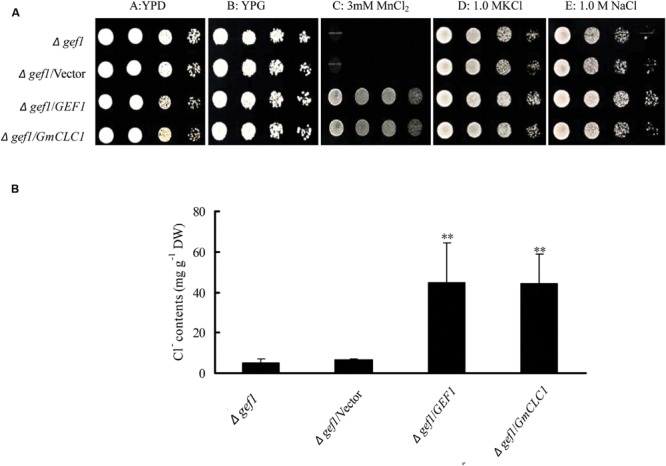
**Effects of expressing *GmCLC1* on the survival of the *Δgef1* yeast mutant under MnCl_2_, NaCl, or KCl. (A)** *GmCLC1* could enhance the survival of the chloride channel-deficient *Δgef1* yeast mutant in the same way as *GEF1*, when the yeast was grown in media supplemented with MnCl_2_, KCl, and NaCl. The *Δgef1* mutant was also transformed with an empty vector as a negative control. The expression vector pYES2 containing *GEF1* or *GmCLC1* was introduced into the *Δgef1* mutant. Ten-fold serial dilutions (starting at OD_550_ ≈ 0.5) of each sample were plated on media of YPD, YPG, or YPG supplemented with 3 mM MnCl_2_, 1.0 M KCl, or 1.0 M NaCl. Plates were incubated at 30°C and photographs were taken after 60 h. **(B)** The expression of *GmCLC1* in the *gef1* yeast mutant increased Cl^-^ accumulation. Cells of the above-mentioned *Δgef1* mutant, *Δgef1*/*Vector, Δgef1*/*GEF1*, or Δ*gef1*/*GmCLC1* were grown in liquid YPG medium with 1.0 M NaCl. When cultures reached OD_550_ ≈ 0.2, cells were collected by filtration, and their Cl^-^ contents were determined. DW, dry weigh. Data shown are the means and standard deviations of ion contents of three independent cultures of each strain. ^∗∗^: The difference between was significant at *p* < 0.01, using Duncan’s test.

All the yeast strains, including the mutant *Δgef1* (a *GEF1*-deficient mutant), and *Δgef1* transformed with empty vector, *GEF1*, and *GmCLC1*, grew well on both YPD and YPG media under normal growing conditions. When cultured on YPG medium supplemented with 3 mM MnCl_2_, the mutant *Δgef1* and *Δgef1*/Vector were unable to grow, but the mutants transformed with yeast *GEF1* or soybean *GmCLC1* could. Similarly, mutants transformed with yeast *GEF1* or soybean *GmCLC1* grew better on YPG medium supplemented with 1.0 M NaCl or 1.0 M KCl (**Figure 6A**). The Cl^-^ contents in the cells of *Δgef1*/*GEF1* and *Δgef1*/*GmCLC1* grown in liquid YPG medium plus 1.0 M NaCl were significantly higher than in the control cells without intracellular vesicle-localized *GEF1* transporter activity *(Δgef1* and *Δgef1*/Vector; *p* < 0.01; **Figure 6B**). Thus *GmCLC1* may have similar functions much like the yeast *GEF1*.

## Discussion

Ionic toxicity is the main cause of salt injury for plants or crops, and Cl^-^ is the main culprit. The predominant strategies for plants to reduce the effects of salt stress are via active Cl^-^ eﬄux or vacuolar Cl^-^ partitioning to reestablish intracellular Cl^-^ homeostasis (Tealle and Tyerman, 2010; Wei et al., 2013; Wong et al., 2013), especially for chloride-intolerant plants such as the cultivated soybean, citrus, grape, potato, tobacco, and so on (Moya et al., 2003; Henderson et al., 2014).

In the *Arabidopsis* genome, a total of seven genes (*AtCLCa-g*) encoding putative CLC proteins have been identified. For example, *AtCLCc* is mainly expressed in stomatal guard cells, and the AtCLCc protein located in the tonoplast displays transmembrane Cl^-^-transporting activity, which not only aids in the regulation of stomatal movements, but also in enhancing salt tolerance (Lv et al., 2009; Jossier et al., 2010). Nguyen et al. (2015) reported that a knock-out mutant AtCLCg, a member of the *Arabidopsis* CLC family localized in the vacuolar membrane, showed a decrease in biomass and an accumulation of chloride in shoots when grown under NaCl stress. *AtCLCg* was expressed in mesophyll cells, hydathodes and phloem while AtCLCc (with 62% similarity to AtCLCg protein) was expressed in stomata. A *atclcc/atclccg* double mutant was not more sensitive to NaCl than the single mutants, which demonstrated that AtCLCc and AtCLCg formed part of a regulatory network controlling chloride sensitivity and were both important for chloride tolerance but not redundant.

The soybean genome has eight *CLC* genes in total: *Glyma01g44950, Glyma05g14760 (GmCLC1), Glyma09g28620, Glyma11g00690, Glyma13g23080, Glyma16g06190, Glyma16g33351*, and *Glyma19g25680* located on chromosomes 1, 5, 9, 11, 13, 16, and 19, respectively (Li et al., 2014).

We previously reported that *GmCLC1* encodes a putative Cl^-^/H^+^ antiporter (Wong et al., 2013) that is localized on tonoplast (Li et al., 2006). Protective functions of *GmCLC1* were only shown using transgenic tobacco cells (Li et al., 2006). In this study, we conducted functional tests *in planta*.

Our results showed that the ectopic expression of *GmCLC1* in *Arabidopsis* (**Figure 1A**) significantly enhanced the transgenic seeds germination rate and subsequent seedling growth (**Figures 1B–D**), and the transgenic plants were better able to adapt to NaCl stress (**Figure 2A**). Moreover, the alleviation of salt injuries to *GmCLC1*-transgenic *Arabidopsis* plants was correlated with reduction in Cl^-^ transport and accumulation in shoots compared to WT plants (**Figure 3B**). Since *GmCLC1* is tonoplast-localized, it is unlikely that this transporter will exclude Cl^-^ to prevent accumulating of this ion. Therefore, the root Cl^-^ concentration did not differ significantly by expressing *GmCLC1* (**Figure 3A**). In contrast, since *GmCLC1* may help to compartmentalize Cl^-^ into vacuole, it could delay Cl^-^ transporting from root to shoot and hence lead to a lower Cl^-^ concentration in the aerial part, in both transgenic *A. thaliana* (**Figure 3B**) and composite soybean plants (**Figure 5D**).

In a previous study Ali et al. (2012), it was found that over-expression of nine TFs (such as *GmWRKY, GmNAC2, GmbZIP110*, and *GmMYB92*) in hairy roots could enhance the survival or tolerance to 200 mM NaCl stress in mosaic or composite soybean plants. We also demonstrated that cotyledon hairy roots could be used as a rapid gain-of-function test for ion transporters (Qi et al., 2014). In this study, we employed soybean cotyledon hairy roots transformed with *GmCLC1* to investigate the role of this chloride transporter in enhancing salt tolerance (**Figures 4B–D**). We found that overexpression of *GmCLC1* in Jackson or Lee68 hairy root composite plants increased the Cl^-^ sequestering in roots, and, as a consequence, reduced Cl^-^ transportation and accumulation in aerial parts, and alleviated their salt injuries as represented by the REL values in roots and various stages of leaf development. This was especially true for the salt-tolerant Lee68 cultivar (**Figure 5**). All these findings inferred that in *GmCLC1* plays a vital role in enabling the soybean plant to adapt to chloride/salt stress.

To provide more understanding on how the GmCLC1 protein may function to control Cl^-^ accumulation, we made use of a yeast system. The yeast *GEF1* gene encodes a CLC-type chloride channel protein, which is co-localized with Ccc2 proteins to control Cu^2+^ in the intracellular vesicles, and drives Cl^-^ and H^+^ transmembrane exchanges. Thus the yeast GEF1 protein is involved in the co-transport of Cu^2+^ or Fe^3+^, the regulation of cation homeostasis and the growth of yeast cells. In the *Δgef1* loss-of-function yeast mutant, the Cl^-^ transport into the intracellular vesicles (vacuole or Golgi apparatus) was blocked, resulting in hypersensitivity to extracellular cations such as Cu^2+^, Fe^3+^, and Mn^2+^, hygromycin B, tetramethyl ammonium chloride, and thus growth were hindered (Gaxiola et al., 1998; Lv et al., 2009; Sasvari et al., 2013). Among the seven *Arabidopsis CLC* paralogs (*AtCLCa-g*), over-expressions of *AtCLCa, AtCLCd*, and *AtCLCf* in the yeast *gef1* mutant could complement the deficiency in GEF1 protein functions or growth phenotype (Lv et al., 2009; Barbier-Brygo et al., 2011). When the soybean *GmCLC1* or the yeast *GEF1* gene was transformed into the *Δgef1* mutant and cultured in YPG media containing different chloride salts (MnCl_2_, KCl, NaCl), the growth of the transformed mutants was much enhanced compared to the original mutant. Therefore, *GmCLC1* may help restore the Cl^-^ transportation into intercellular vesicles.

## Author Contributions

PW and LW conducted the experiments, collected and analyzed all data. BY and H-ML designed the experiments. BY, H-ML, and AL interpreted the data and wrote the manuscript. All authors read and approved the final version of the manuscript.

## Conflict of Interest Statement

The authors declare that the research was conducted in the absence of any commercial or financial relationships that could be construed as a potential conflict of interest.

## Supplementary Material

FIGURE S1 **Growth characteristics of *GmCLC1*-transgenic *Arabidopsis thaliana*. (A)** Growth of WT and transgenic *Arabidopsis GmCLC1* (L_1_–L_5_) under salt stress. Comparison in **(B)** fresh weight (FW) and **(C)** shoot fresh weight (FW) of WT and *GmCLC1*-transgenic *Arabidopsis* plants (L_1_, L_2_, L_5_). Seeds of WT and *Arabidopsis* transgenic *GmCLC1* plants were surface-sterilized and kept at 4°C for 2–4 days and then sown on MS medium. After 8 days of incubation, the seedlings were transferred into plastic pots filled with a sterilized peat moss and vermiculite mixture, and fertilized with 1/2-strength Hoagland nutrient solution for 5 days in the greenhouse. The nutrient solution was continuously replaced with half-strength Hoagland solution containing 50 mM NaCl for 2 days, followed by 100 mM NaCl for 2 days, and finally 150 mM NaCl for 6 days. No NaCl was added to the nutrient solution for the control treatment. ^∗^/^∗∗^: The difference between WT and *GmCLC1* was significant at *p* < 0.05/0.01, respectively, using Duncan’s test. Each bar represents the mean and SD of at least three replicates.
