# Supplementary figures and images for: GmCLC1 Confers Enhanced Salt Tolerance through Regulating Chloride Accumulation in Soybean
Source: Front Plant Sci. 2016 Jul 25;7:1082. doi: 10.3389/fpls.2016.01082 (PMC4959425; doi:10.3389/fpls.2016.01082)

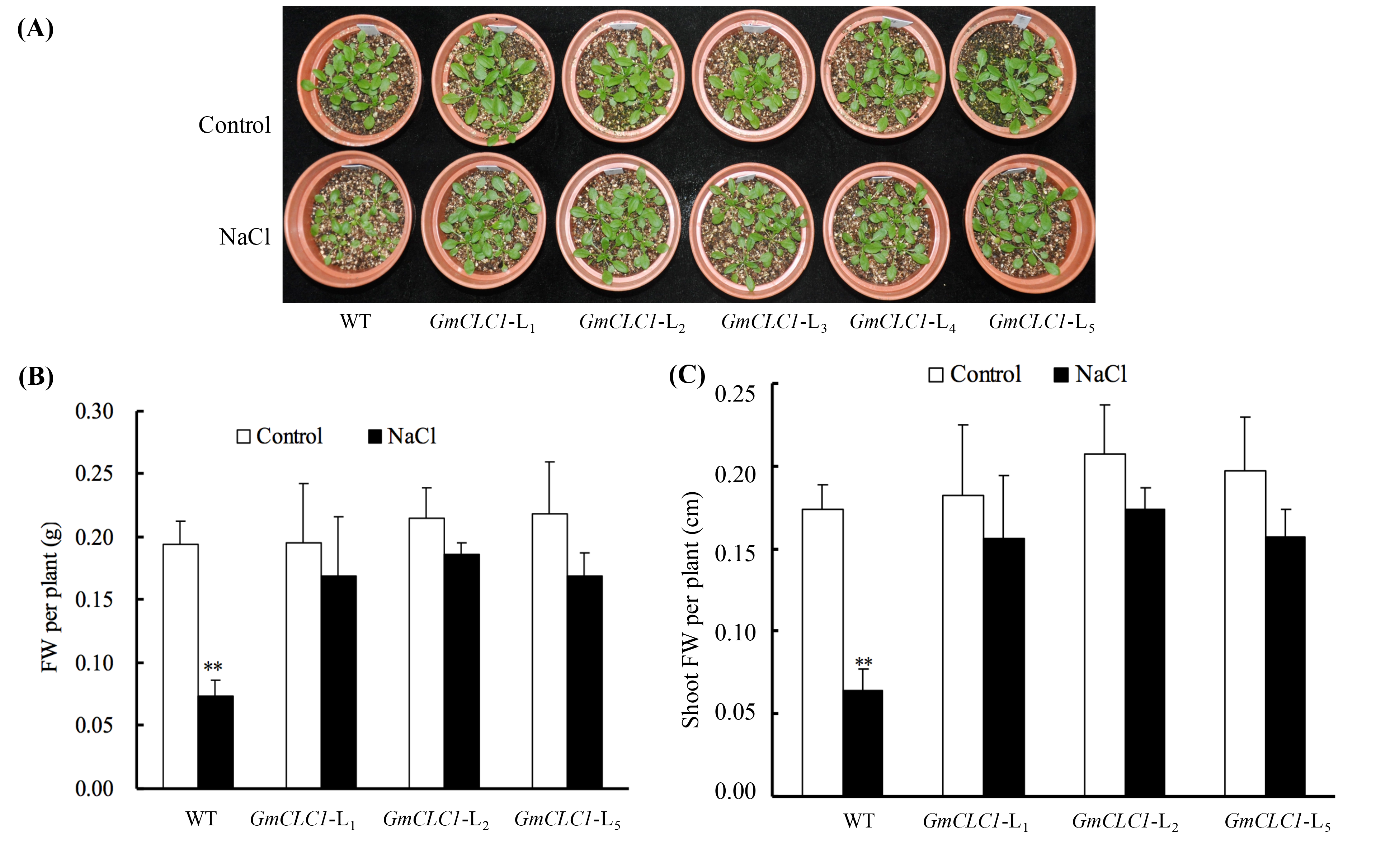

Supplement: FIGURE S1 — Growth characteristics of GmCLC1-transgenic Arabidopsis thaliana. (A) Growth of WT and transgenic Arabidopsis GmCLC1 (L1–L5) under salt stress. Comparison in (B) fresh weight (FW) and (C) shoot fresh weight (FW) of WT and GmCLC1-transgenic Arabidopsis plants (L1, L2, L5). Seeds of WT and Arabidopsis transgenic GmCLC1 plants were surface-sterilized and kept at 4°C for 2–4 days and then sown on MS medium. After 8 days of incubation, the seedlings were transferred into plastic pots filled with a sterilized peat moss and vermiculite mixture, and fertilized with 1/2-strength Hoagland nutrient solution for 5 days in the greenhouse. The nutrient solution was continuously replaced with half-strength Hoagland solution containing 50 mM NaCl for 2 days, followed by 100 mM NaCl for 2 days, and finally 150 mM NaCl for 6 days. No NaCl was added to the nutrient solution for the control treatment. ∗/∗∗: The difference between WT and GmCLC1 was significant at p < 0.05/0.01, respectively, using Duncan’s test. Each bar represents the mean and SD of at least three replicates. [file Image_1.TIF]
